# Discovery of long non-coding RNAs in *Aspergillus flavus* response to water activity, CO_2_ concentration, and temperature changes

**DOI:** 10.1038/s41598-023-37236-4

**Published:** 2023-06-26

**Authors:** Nafiseh Davati, Abozar Ghorbani

**Affiliations:** 1 Department of Food Science and Technology, College of Food Industry, Bu-Ali Sina University, Hamedan, 65167-38695 Iran; 2 Nuclear Agriculture Research School, Nuclear Science and Technology Research Institute (NSTRI), Karaj, Iran

**Keywords:** Fungi, Computational biology and bioinformatics, Genetics, Microbiology, Molecular biology

## Abstract

Although the role of long non-coding RNAs (lncRNAs) in key biological processes in animals and plants has been confirmed for decades, their identification in fungi remains limited. In this study, we discovered and characterized lncRNAs in *Aspergillus flavus* in response to changes in water activity, CO_2_ concentration, and temperature, and predicted their regulatory roles in cellular functions. A total of 472 lncRNAs were identified in the genome of *A. flavus*, consisting of 470 novel lncRNAs and 2 putative lncRNAs (EFT00053849670 and EFT00053849665). Our analysis of lncRNA expression revealed significant differential expression under stress conditions in *A. flavus*. Our findings indicate that lncRNAs in *A. flavus*, particularly down-regulated lncRNAs, may play pivotal regulatory roles in aflatoxin biosynthesis, respiratory activities, cellular survival, and metabolic maintenance under stress conditions. Additionally, we predicted that sense lncRNAs down-regulated by a temperature of 30 °C, osmotic stress, and CO_2_ concentration might indirectly regulate proline metabolism. Furthermore, subcellular localization analysis revealed that up-and down-regulated lncRNAs are frequently localized in the nucleus under stress conditions, particularly at a water activity of 0.91, while most up-regulated lncRNAs may be located in the cytoplasm under high CO_2_ concentration.

## Introduction

*Aspergillus flavus* is a fungal species in the family *Trichocomaceae*, and it is a saprotrophic and opportunistic human pathogen^1^. It is also known as a postharvest rot pathogen, causing crop spoilage during harvest, storage, and transportation^2^. *A. flavus* can produce toxic secondary metabolites, including aflatoxins, which are the most carcinogenic and mutagenic mycotoxins produced by several fungal species, mainly *A. flavus*, and cause disease and death in animals and humans. Chronic exposure to AFB1 has been associated with various disorders in humans and animals^3^. The International Agency for Research on Cancer (IARC) has designated aflatoxins, particularly AFB1, as Class 1A human carcinogens. Mycotoxins, especially aflatoxins, are considered a serious threat to the food industry, and many countries impose strict controls and stringent regulatory limits on a wide range of food and feed products^4^. Temperature, CO_2_ concentration, and water activity (a_w_) are among the abiotic factors that affect fungal growth and the production of secondary metabolites, including mycotoxins^5–8^. *A. flavus* has adapted to stressful environmental conditions by enhancing its physiological systems to dominate other microbial communities^9,10^. Recent advances in RNA-seq technology have allowed for a better understanding of the role of different gene groups in resistance to abiotic stresses and their interactions. Non-coding RNAs (ncRNAs) are RNA molecules that are not translated into proteins, including long non-coding RNAs (lncRNAs) and small ncRNAs (sncRNAs)^11,12^. SncRNAs, ranging from 18 to 200 nt in length, play key roles in regulating cellular functions, whereas lncRNAs, molecules longer than 200 nt, regulate gene expression at various levels (transcription, RNA processing, translation, and post-translation) by interacting with proteins and nucleotides^11,12^. LncRNAs are more complex than sncRNAs and have adaptive regulatory activities in various biological processes, such as alternative splicing, X-chromosome inactivation, dosage compensation, and genomic imprinting^13–18^. LncRNAs have been categorized as antisense, sense, intronic, bidirectional, and intergenic based on their relative positions to neighboring coding genes. Certain lncRNAs can be translated into micro-peptides or serve as sponges for microRNA recruitment^19–23^. Some lncRNAs in the form of circular RNAs (circRNAs) function as transcriptional effectors that control target gene expression^24–27^. Expression of regulatory lncRNAs often occurs at specific developmental stages or in response to changes in diet or environmental conditions^13,15,28,29^. In this study, we discovered lncRNAs in the response of *A. flavus* to changes in a_w_, CO_2_ concentration, and temperature and describe their regulatory functions in regulating gene expression and their interaction with milRNAs targets. In fungi, mature miRNAs are produced from miRNA precursors by Dicer enzymes^30^; thus, fungal miRNAs should be referred to as microRNA-like RNAs (milRNAs). MilRNAs play important roles in regulating fungal gene expression and pathogenicity mechanisms under different environmental conditions^30–32^. The predicted targets of lncRNAs demonstrate their potential regulatory roles and the study of lncRNAs related to aflatoxin biosynthesis and fungal metabolism may enhance our understanding of the regulatory functions of lncRNAs under different stress conditions. Given the importance of the presence of *A. flavus* and its mycotoxins in food products, this study could have significant implications for improving food safety and quality.

## Results and discussion

In this study, 22 raw RNA-Seq data from *A. flavus* were used and the quality control results are shown in Supplementary File 1.

### Discovery and characterization of *A. flavus* lncRNAs

Numerous studies have demonstrated that long non-coding RNAs (lncRNAs) play key roles in biological processes, particularly in mammals. In fungi, the roles of lncRNAs have also been reported in cellular development, pathogenicity, regulation of biological processes, and metabolism^12^. However, to date, there have been no reports on the discovery of lncRNAs in *A. flavus*, with most studies being conducted on *Saccharomyces cerevisiae*. Table 1 lists the lncRNAs that have been experimentally characterized in fungal cells.

**Table 1:** Discovered and identified lncRNAs of fungal cells.

Microorganism	lncRNAs
*S. cerevisiae*	GAL10-ncRNA^33^, GAL4 lncRNA^34^, ncASP3^35^, PHO84 antisense transcripts^36^, REM2^37^, IRT1^38^, REM3^39^, SRG1^40^, pHO-lncRNA^41^, PWR1, ICR1^42^, AS-PHO5 ^43^, Antisense lncRNA of CDC28^44^, Antisense lncRNA of Ty1^45^, TERRA^46^, TLC1^47^, ADF1^48^
*Schizosaccharomyces pombe*	prt/nc-pho1^49^, nc-tgp1^50^, prt2 ^51^, mlonRNAs^52^, SPNCRNA 1164^53^, meiRNA-S and L^54^, TER1^55^
*Trichoderma reesei*	HAX1^56^
*Cochliobolus heterostrophus*	Antisense of transcription factor CMR1
*Neurospora crassa*	qrf^57^
*Ustilago maydis*	Antisense to gene um02151^58^
*Cryptococcus neoformans*	RZE1^59^
*Fusarium oxysporum*	Fo-carP and Ff-carP^60^

In the present study, a total of 472 long non-coding RNAs (lncRNAs) were identified in the genome of *A. flavus* in response to changes in water activity (a_w_), CO_2_ concentration, and temperature.

The minimum, average, and maximum lengths of lncRNAs were calculated to be 250, 393.625, and 3679, respectively. Among the identified lncRNAs, 470 were classified as novel, while the remaining 2 were categorized as putative. The putative lncRNAs, namely EFT00053849670 and EFT00053849665, were found to correspond to the 28S ribosomal RNA (G4B11_011282) and 18S ribosomal RNA (G4B11_011297) genes, respectively. These lncRNAs were located within chromosome 7. Notably, no scientific literature on these particular genes could be found in published articles or the Europe PubMed Central database.

### Differential expression of lncRNAs

**Figure 1:**
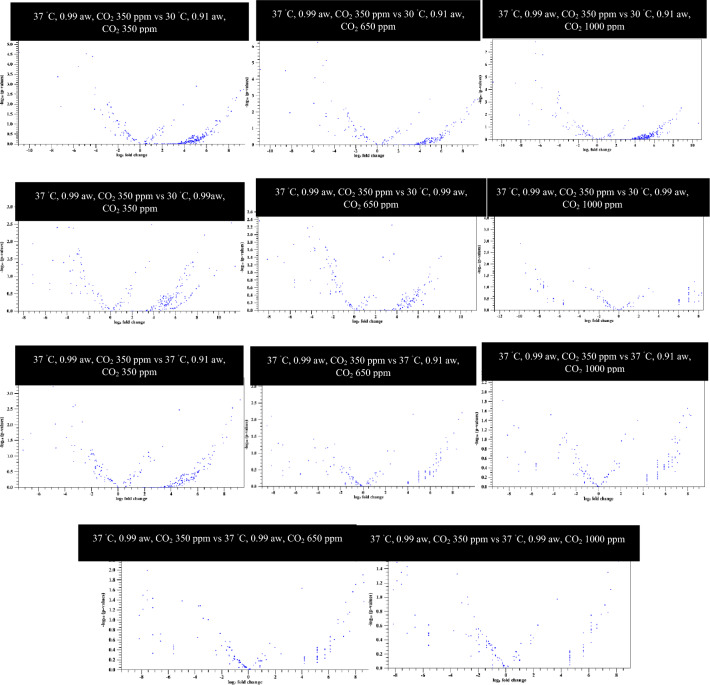
Volcano plot of differentially expressed lncRNAs in treatments compared with control (37 °C, a_w_ = 0.99, CO_2_ = 350 ppm).

**Figure 2:**
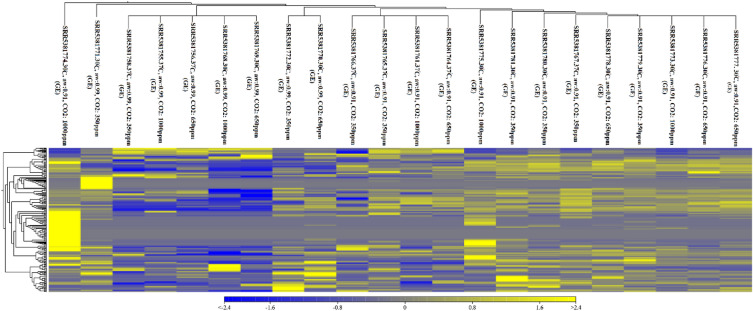
Heatmap of differentially expressed lncRNAs in treatments compared with control (37 °C, a_w_: 0.99, CO_2_: 350 ppm). (Up-regulated lncRNAs were labeled in yellow, and down-regulated lncRNAs were labeled in blue).

In this study, the data belonging to the optimal growth conditions (37 °C, a_w_: 0.99, and CO_2_: 350 ppm) were considered as the control group. The expression of the other treatments (experimental groups) was compared with the control group expression, as scientific reports have confirmed that the optimal growth of *A. flavus* occurs under the conditions defined for the control^61–66^. The detailed information on the top 10 up- and down-regulated lncRNAs of *A. flavus* in response to changes in a_w_, CO_2_ concentration, and temperature are summarized in supplementary files 2 and 3. Volcano plots (Fig. 1) and hierarchical cluster analysis (Fig. 2) demonstrated differential expression of lncRNAs in the treatments compared with the control. The results showed that the highest fold changes of the up-regulated lncRNAs occurred in the treatments with a temperature of 30 °C, a_w_: 0.99, CO_2_: 350 ppm (FC: 2317.40) and 30 °C, a_w_: 0.99, CO_2_: 650 ppm (FC: 2277.39). The lowest changes occurred in the treatments with a temperature of 30 °C, a_w_: 0.99, CO_2_: 1000 ppm (FC: −4199.28); 30 °C, a_w_: 0.91, CO_2_: 1000 ppm (FC: −1935.04); 30 °C, a_w_: 0.91, CO_2_: 650 ppm (FC: −1935.04); and 30 °C, a_w_: 0.91, CO_2_: 350 ppm (FC: −1935.04). Based on the volcano plots, the number of up-regulated lncRNAs relative to down-regulated lncRNAs is higher in most treatments. Figure 1 shows that with increasing CO_2_ concentration, the number of both up-and down-regulated lncRNAs was reduced in most treatments at the same temperature and a_w_. With decreasing a_w_, the number of up-regulated lncRNAs increased in most treatments at the same temperature and CO_2_ concentration, especially at CO_2_: 1000 ppm and 30 °C. According to Fig. 2, the number of up-regulated lncRNAs increased under temperature and a_w_ stress, with the most up-regulated lncRNAs found in the treatments with a temperature of 30 °C and a_w_: 0.91. With increasing a_w_, the number of down-regulated lncRNAs also increased. Thus, the up-regulation of lncRNAs was more influenced by low water activity (a_w_), and their down-regulation was more influenced by high water activity. A previous study showed that an increase in temperature from 30 to 37 °C resulted in differential expression of 2224 genes at a_w_: 0.99 and 481 genes at a_w_: 0.91. Subsequently, 12 biological processes were up-regulated, and 9 were down-regulated^64^. Following Medina et al.^64^, the results of our study indicate that the down-regulation of the discovered lncRNAs may play a regulatory role in the growth and metabolism of *A. flavus* under optimal growth conditions. Additionally, the up-regulation of the discovered lncRNAs affected by low a_w_ may play important functions in the down-regulation of genes of *A. flavus* at a_w_: 0.91. Several studies have shown that many lncRNAs were differentially expressed by fungi due to environmental stress. According to Nadal-Ribelles et al.^44^, a large number of lncRNAs from *S. cerevisiae* were up-regulated under osmotic stress, such as the antisense lncRNA of CDC28. This lncRNA was defined as a cis-regulator of the master cell cycle regulator CDK1/Cdc2 induced by Hog1. In *S. pombe*, the lncRNA PNCRNA.1164 (with unknown mode) was also identified as a trans-regulator of a stress-responsive transcription factor, Atf1, exposed to oxidative stress^53^. Metabolic stress-induced lncRNAs, such as the antisense lncRNA of the CDC28 gene and ncASP3 from *S. cerevisiae*, and mlonRNA (mRNA-like long ncRNAs^67^) in *S. pombe*, are involved in stress responses through the regulatory mechanism of chromatin remodeling and organization^44^. Figure 2 shows that CO_2_ concentration had different effects on the expression of lncRNAs. With increasing CO_2_ concentration, the number of up-regulated lncRNAs increased at 30 °C and a_w_: 0.91, whereas with increasing CO_2_ concentration, the number of down-regulated lncRNAs increased at 30 °C and a_w_: 0.99. As mentioned earlier (Fig. 1), high water activity can cause the number of down-regulated lncRNAs to increase, and vice versa. Among the top differentially expressed lncRNAs, which were shared in most treatments (Table 2), lnc59 probably plays an important regulatory role in *A. flavus* growth under stress conditions because its expression decreased at all levels of stressors. Additionally, other important lncRNAs, such as lnc124, lnc85, and lnc43, may be responsible for important regulatory functions in *A. flavus* under stress conditions.

**Table 2:** Common significant lncRNAs in the response of *A. flavus* to temperature, CO_2_ concentrations, and a_w_ stress.

Feature ID	Fold change of up-regulated lncRNAs in a_w_ = 0.91,* P*-value (0.05)					
	30 °C, a_w_ = 0.91, CO_2_ 350 ppm	30 °C, a_w_ = 0.91, CO_2_ 650 ppm	30 °C, a_w_ = 0.91, CO_2_ 1000 ppm	37 °C, a_w_ = 0.91, CO_2_ 650 ppm	37 °C, a_w_ = 0.91, CO_2_ 1000 ppm	37 °C, a_w_ = 0.91, CO_2_ 350 ppm
lnc124	532.68	561.68	458.45	867.00	539.31	590.74
lnc85	327.86	519.99	419.77	183.316	250.93	394.93

Feature ID	Fold change of down-regulated lncRNAs in a_w_ = 0.91,* P*-value (0.05)					
	30 °C, a_w_ = 0.91, CO_2_ 350 ppm	30 °C, a_w_ = 0.91, CO_2_ 650 ppm	30 °C, a_w_ = 0.91, CO_2_ 1000 ppm	37 °C, a_w_ = 0.91, CO_2_ 650 ppm	37 °C, a_w_ = 0.91, CO_2_ 1000 ppm	37 °C, a_w_ = 0.91, CO_2_ 350 ppm
lnc43	− 48.12	− 378.37	− 378.37	− 378.37	− 378.37	− 76.99
lnc59	− 16.53	− 32.98	− 17.67	− 21.073	− 661.40	− 27.69

Feature ID	Fold change of down-regulated lncRNAs at 30 °C,* P*-value (0.05)					
	30 °C, a_w_ = 0.91, CO_2_ 350 ppm	30 °C, a_w_ = 0.91, CO_2_ 650 ppm	30 °C, a_w_ = 0.91, CO_2_ 1000 ppm	30 °C, a_w_ = 0.99, CO_2_ 350 ppm	30 °C, a_w_ = 0.99, CO_2_ 650 ppm	30 °C, a_w_ = 0.99, CO_2_ 1000 ppm
lnc59	− 16.53	− 32.98	− 17.67	− 31.65	− 39.24	− 661.40

Feature ID	Fold change of down-regulated lncRNAs in CO_2_ concentration = 650 ppm,* P*-value (0.05)					
	30 °C, a_w_ = 0.91, CO_2_ 650 ppm	30 °C, a_w_ = 0.99, CO_2_ 650 ppm	37, a_w_ = 0.91, CO_2_ 650 ppm	37 °C, a_w_ = 0.99, CO_2_ 650 ppm		
lnc59	− 32.98	− 39.24	− 21.07	− 661.40		

### Prediction of potential target genes of lncRNAs

**Figure 3:**
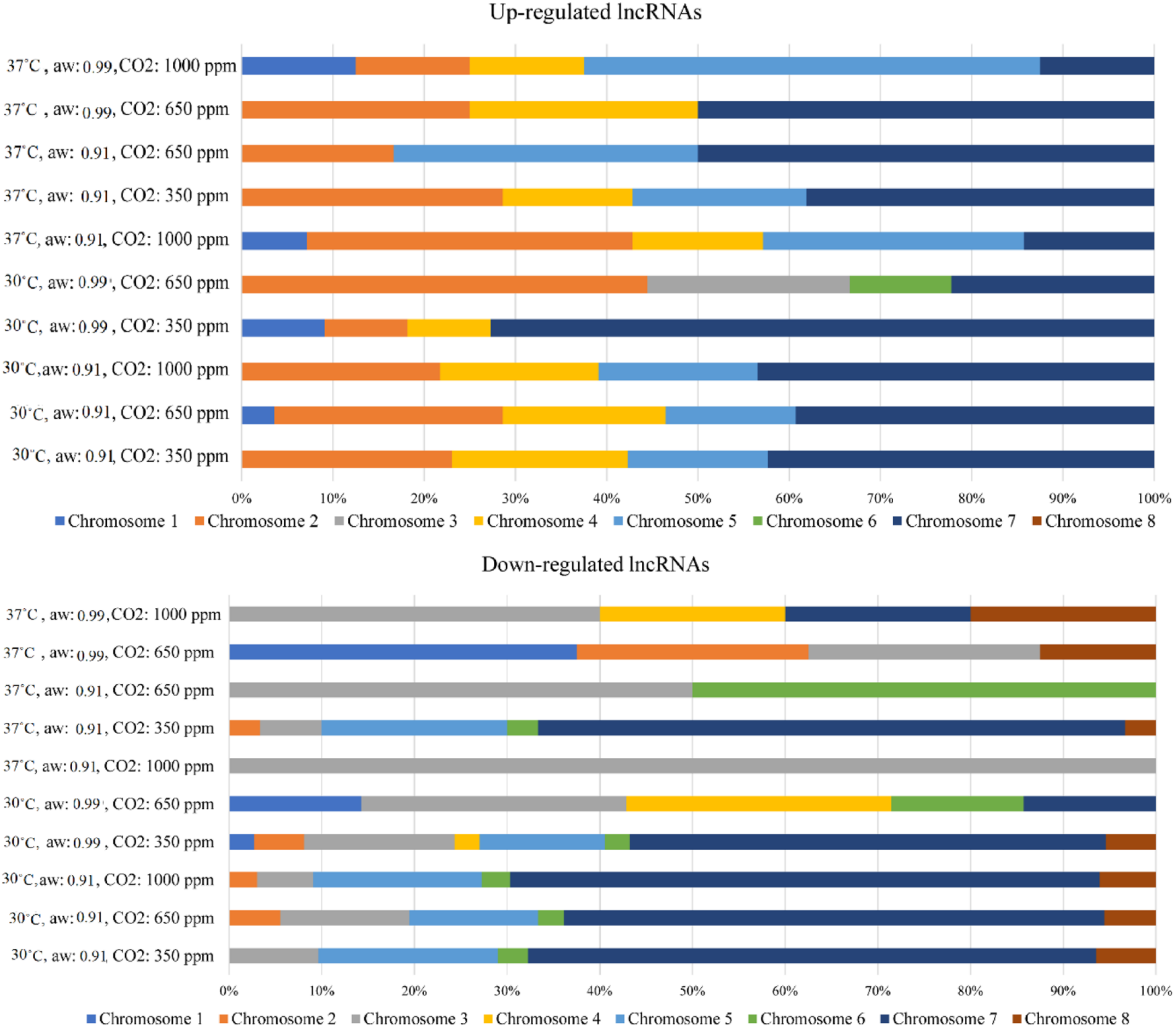
Up- and down-regulated lncRNAs under stress conditions located on different chromosomes compared with control (37 °C, a_w_ = 0.99, CO_2_ = 350 ppm).

As shown in Fig. 3, most of the up-and down-regulated lncRNAs were located on chromosome 7. Therefore, to identify the regulatory function of the differentially expressed lncRNAs, their target genes were predicted within a range of 10,000 bp only on this chromosome. In the current study, it was predicted that the down-regulated lncRNAs from 5 treatments targeted similar genes on chromosome 7. These treatments included 30 °C, a_w_: 0.91, CO_2_: 350 ppm; 30 °C, a_w_: 0.91, CO_2_: 650 ppm; 30 °C, a_w_: 0.91, CO_2_: 1000 ppm; 30 °C, a_w_: 0.99, CO_2_: 350 ppm; and 37 °C, a_w_: 0.91, CO_2_: 350 ppm. These lncRNAs likely induce genes associated with optimal growth of *A. flavus*, as they were more down-regulated at low temperatures and osmotic stress. The genes identified in these regions that were affected by the regulatory mechanism of lncRNAs include F9C07_1908990, F9C07_1910648, F9C07_2062407, F9C07_13346, and F9C07_13347. F9C07_1908990 and F9C07_1910648 are related to the production of a hypothetical protein that is predicted to be expressed in fungal cells but could not be experimentally demonstrated to exist. Several hypothetical proteins have been reported to be associated with *A. flavus* metabolism and aflatoxin biosynthesis, and some of them are produced under environmental stress, such as temperature^68^. The gene F9C07_2062407, delta-1-pyrroline-5-carboxylate dehydrogenase, is involved in glutamate production through the proline cycle and is up-regulated under osmotic and oxidative stress^69,70^. In addition, the expression of F9C07_13346 and F9C07_13347 results in putative pyrroline-5-carboxylate reductase activity involved in the proline biosynthesis process^71^.

Proline serves as an energy source in eukaryotic cells to maintain cell structure and support cell growth following environmental stress^72,73^. According to Lemieux and Blier, temperature changes affect oxidative phosphorylation pathways for cellular compatibility, including proline dehydrogenase, sulfide quinone oxidoreductase, the electron-transferring flavoprotein pathway, dihydroorotate dehydrogenase, glycerophosphate dehydrogenase, and choline dehydrogenase^74^.

Hamayun et al. also indicated that proline metabolism was associated with tolerance to temperature changes in *A. flavus*^75^.

Proline dehydrogenase (ProDH) causes the oxidation of L-proline to pyrroline-5-carboxylate and is located in the inner membrane of mitochondria^76^. It has been reported that ProDH protects cellular respiration under stressful conditions, and proline and ProDH reduce the effects of oxidative stress by maintaining NADPH^77,78^. In general, proline metabolism in *A. flavus* is important at different temperatures, while fungal growth and aflatoxin production are affected by the down-regulation of proline oxidase^68^. Therefore, it was predicted that the down-regulated lncRNAs affected by the temperature of 30 °C, osmotic stress, and CO_2_ concentrations might indirectly regulate proline metabolism.

To date, 30 genes (referred to as toxin-producing gene cluster) involved in aflatoxin biosynthesis in *A. flavus* have been identified, and all of them are located on chromosome 3^79,80^.

Moreover, in the current study, the down-regulated lncRNAs were only located on chromosome 3 under stress conditions of 37 °C, aw = 0.91 and CO2 = 1000 ppm, where the aflatoxin biosynthesis cluster is located. Additionally, the down-regulated lncRNAs found from other treatments were also located on chromosome 3.

Medina et al. indicated that several genes related to aflatoxin production were down-regulated under 30 °C, a_w_ = 0.99, compared with the control (37 °C, a_w_ = 0.99), including aflY, aflX, aflV, aflK, aflP, aflO, aflL, aflG, aflN, aflM, aflE, aflJ, aflH, aflS, aflR, aflB, aflT, and aflU^64^.

Similarly, in the current analysis, some down-regulated lncRNAs found at 30 °C and aw = 0.99 were located on chromosome 3. The correlation between most down-regulated lncRNAs and down-regulated genes in the aflatoxin-biosynthesis gene cluster showed a negative correlation with these genes.

### Predicted interactions between lncRNAs and milRNAs

Three classes of sncRNAs, including small interfering RNAs (siRNAs), microRNAs (miRNAs), and PIWI-interacting RNAs (piRNAs), have been associated as negative regulators of the expression of target RNAs that regulate several physiological and cellular processes^81–85^. The functions of sncRNAs have been found in several filamentous fungi^86,87^, but few studies have been performed to identify miRNAs (or milRNAs in fungi) and their functions. It should be noted that the milRNA pathway is an ancient and protected regulatory system in multicellular microorganisms, so the milRNAs of *A. flavus* may be important for fungal growth and aflatoxin biosynthesis^88^.

**Figure 4:**
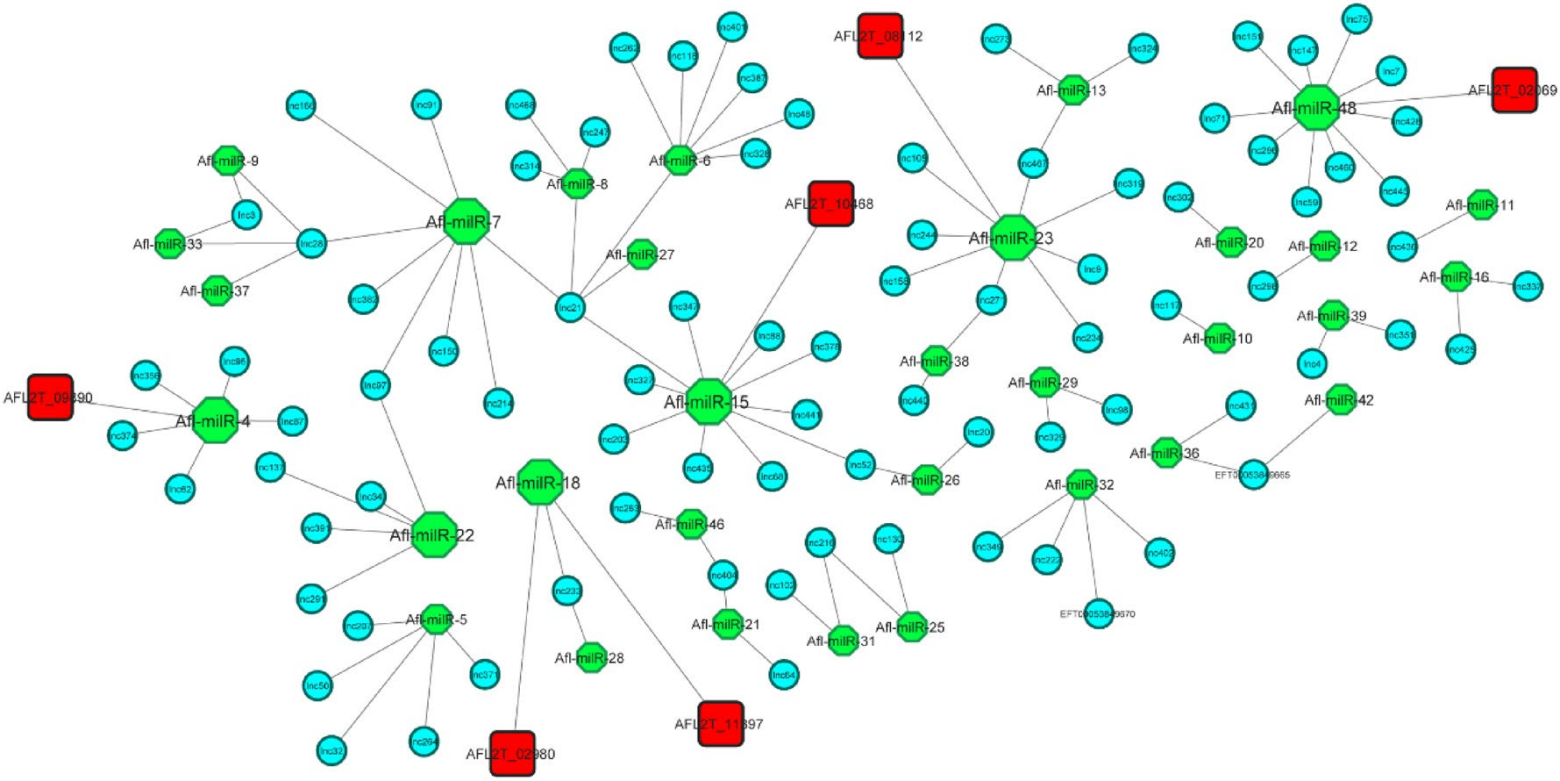
The network of predicted relationships among lncRNAs, target milRNAs, and proteins in *A. flavus* under stress conditions. LncRNAs: blue circle, Hub milRNAs: large green octagon, Target proteins: red square.

To investigate whether the lncRNAs discovered in this study could target milRNAs and subsequently genes in *A. flavus*, target milRNAs of lncRNAs and target genes of milRNAs were predicted^66,88,89^, and the predicted links between them are shown in Fig. 4. In general, among the significantly down-regulated lncRNAs, 11 lncRNAs, including EFT00053849670, EFT00053849665, lnc4, lnc9, lnc21, lnc48, lnc50, lnc52, lnc59, lnc62, and lnc216, were associated with 10 different milRNAs. Among the significantly up-regulated lncRNAs, only 3 lncRNAs, including lnc3, lnc52, and lnc216, were associated with 3 different milRNAs. As a result, it can be inferred that milRNAs and subsequently proteins in *A. flavus* are more affected by down-regulated lncRNAs than up-regulated lncRNAs under stress conditions. Detailed information on the top lncRNAs in this analysis can also be found in Supplementary File 4.

Among the 17 milRNAs, only seven milRNAs (Afl-milR-48, Afl-milR-15, Afl-milR-7, Afl-milR-23, Afl-milR-18, Afl-milR-22, and Afl-milR-4) were identified as hub milRNAs (Table 3, Fig. 4). The results of predicted interactions of hub-milRNAs with target proteins of *A. flavus* showed that the target genes of Afl-milR-48 and Afl-milR-4 encode hypothetical proteins. In general, of the total lncRNAs discovered, 35 lncRNAs were predicted to bind to 7 milRNAs linked to 10 hypothetical proteins.

**Table 3:**
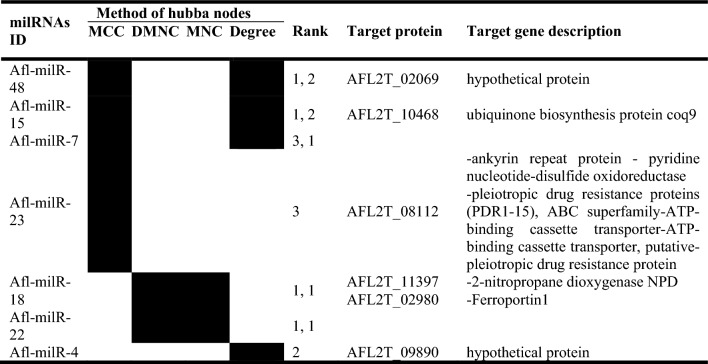
Hub milRNAs of *A. flavus* interacted with target proteins.

The Rank values generated by cytoHubba represent the relative significance of each gene within the network, with lower numbers indicating higher importance.

According to the previous study, several differentially expressed genes (DEGs) associated with hypothetical proteins were reported in *A. flavus* at 30 °C for the aflatoxin and cyclopiazonic acid clusters. These include aflY/hypA/hypP/hypothetical protein; aflLa/hypB/hypothetical protein; aflMa/hypE/hypothetical protein (a_w_: 0.99); and aflNa/hypD/hypothetical protein (a_w_: 0.99, 0.91)^64^.

Bai et al.^88^ found that the target gene of Afl-milR-4 is expressed at 28 °C, 37 °C, a_w_: 0.93, and a_w_: 0.99 in *A. flavus*. This gene encodes a conserved protein containing an ACT domain (AFL2G_09890), which may play a crucial role in controlling signal transduction, cellular metabolism, and solvent transport. Our results indicate that Afl-milR-4 is associated with the down-regulated lnc62 at a_w_: 0.99, CO_2_: 650 ppm, and both 37 °C and 30 °C. This finding suggests that the expression of lnc62 may regulate functions related to solvent transport, signal transfer, and cellular metabolism under optimal conditions. Furthermore, Afl-milR-48 was associated with the down-regulated lnc59 under all stress conditions, and Afl-milR-33 was associated with two lncRNAs targeted three proteins, including AFL2T_08520, AFL2T_03607 (hypothetical protein), and AFL2T_08912. It was reported that Afl-milR-33 was up-regulated in *A. flavus* at 37 °C and targeted AFL2T_08520 (named B8NM71), which is responsible for gamma-glutamyltranspeptidase (UstH) activity, gamma-glutamyltransferase-selenocysteine lyase activity, and the biosynthesis of ustiloxin B (UstA)^88,90^. Ustiloxin B is a toxic secondary metabolite with a cyclic peptide originally known in *Ustilaginoidea virens* and other fungal species such as *A. flavus*. Two peptidases, UstH and UstP (serine peptidase), have been implicated in the hydrolysis of glutathione associated with ustiloxin B metabolism in *A. flavus*^90^. C-glutamyl transpeptidase hydrolyzes glutathione to glutamate and cysteinyl glycine and can also transfer the c-glutamyl moiety of glutathione to other amino acids^91,92^. Bai et al.^88^ suggested that Afl-milR-33 plays a key role in mycotoxin biosynthesis and temperature-dependent regulation in *A. flavus*.

Considering that lnc3, which is linked to Afl-milR-33, was significantly up-regulated at 37 °C, a_w_: 0.99, and CO_2_: 1000 ppm in the current study, it can be assumed that the expression of lnc3 plays a regulatory role in mycotoxin biosynthesis under high CO_2_ concentrations. In our study, Afl-milR-15 (hub miRNAs) and Afl-milR-6 targeted AFL2T_10468, which is linked to down-regulated lnc21 and both up-and down-regulated lnc52. AFL2T_10468 encodes the ubiquinone biosynthesis protein coq9. Similarly, Afl-milR-15 and Afl-milR-6 targeted AFL2T_09091, which is linked to down-regulated lnc21 and lnc48. AFL2T_09091 encodes cytochrome P450. Our findings demonstrate that oxidative and temperature stresses can affect the expression of mitochondrial-related genes. These genes encode mitochondrial membrane transporters and respiratory enzymes, including cytochrome C oxidase, ubiquinone, and ubiquinol-cytochrome C reductase. Temperature and oxidative stress can also up-regulate genes encoding alternative NADH-ubiquinone oxidoreductase and oxidase (AOX)^66^. Furthermore, different temperatures lead to the down-regulation of Nadh-ubiquinone oxidoreductase kDa mitochondrially in *A. flavus*^68^.

Cytochrome P-450 is known to play a key role in mycotoxin biosynthesis, particularly in the aflatoxin gene cluster, compared to the biosynthetic pathways of other mycotoxins^93^. Additionally, Afl-milR-18 (hub miRNAs) linked to lnc233 targets AFL2T_02980 and AFL2T_11397, which encode 2-nitropropane dioxygenase NPD and ferroportin1, respectively. The aflatoxin biosynthetic pathway involves three bioactive reactions: monooxygenases, dioxygenases (ring cleavage reactions), and Baeyer–Villiger reactions^94,95^. Moreover, ferroptosis, an iron-dependent regulated cell death, is known to occur under stress conditions^96^. Hence, in this analysis, we expected to identify dioxygenases and ferroportin1 as protein targets under stress conditions.

Under stress conditions, aflatoxin biosynthesis and cellular respiratory activities are reduced, especially under high CO_2_ concentration and low a_w_, leading to changes in dioxygenase activity, cytochrome activity, and other related enzymes. Therefore, it is reasonable to speculate that both the up-regulated and down-regulated lncRNAs mentioned above may be involved in the regulation of gene expression. For example, when the ambient CO_2_ concentration increased and cells experienced decreased respiratory activity, lnc21 was down-regulated. Afl-milR-29 was predicted to target AFL2T_01732, which encodes acid histidine phosphatase. This miRNA was associated with lnc98 and lnc329. Histidine metabolism, one of the analysis targets, may be influenced by changes in aromatic amino acid precursors in histidine pathways in response to stress conditions^97^. Among miRNAs, Afl-milR-42 is the target of EFT00053849665 (down-regulated under 30 °C, 37 °C, and low a_w_) and is linked to AFL2T_05705, which encodes a serine-rich protein. Similarly, transcriptome analysis of *A. flavus* revealed that the expression of genes encoding glutamine-serine-proline-rich proteins was up-regulated under different temperatures^68^. Consequently, the down-regulated EFT00053849665 might be related to the regulation of serine-rich protein metabolism under stress conditions. Additionally, other interactions have also been predicted, including Afl-milR-5 (linked to 5 lncRNAs) targeting AFL2T_04761, which encodes palA/RIM20; Afl-milR-12 (linked to 1 lncRNA) targeting AFL2T_09283, which encodes a fungal-specific transcription factor; and Afl-milR-6 (linked to 7 lncRNAs) targeting AFL2T_08349 and AFL2T_10492. It has been reported that the Pal/Rim pathway plays a key role in the survival of fungal cells exposed to environmental stresses such as pH changes^98^. Several upstream regulators related to abiotic stress responses and biosynthesis of secondary metabolites under environmental stress have been identified, whose expression can be regulated by lncRNAs. In response to oxidative stress, several transcription factors have been identified in fungi that activate cellular protective processes against excessive amounts of reactive oxygen species and subsequent damage to proteins, DNA, and lipids. Tian et al. (2021) also reported that the bZIP transcription factors of *atfA* and *atfB*, which regulate aflatoxin biosynthesis, were down-regulated under oxidative and temperature stress. At the same time, the *skn7*-like transcription factor was up-regulated under both oxidative and temperature stress. Furthermore, Li et al.^12^ indicated that lncRNA SPNCRNA.1164 regulates the expression of *Atf1* (a stress-responsive transcription factor), which is affected by oxidative stress. In general, the biosynthesis of aflatoxins occurs via a polyketide pathway involving approximately 22 enzymatic reactions. The genes encoding these enzymes are clustered in the genome, and the expression of key regulatory genes (*aflS; aflR*) and associated structural genes (e.g., *aflD*) is affected by changes in a_w_ and temperature^99,100^. According to Schmidt-Heydt et al.^8^, the level of AFB1 produced by *Aspergillus* spp. correlates significantly with the ratio of *aflR/aflS* expression. The *AflR* protein, as a zinc cluster Zn (II) 2Cys6 transcription factor, binds to several genes related to the aflatoxin cluster, leading to activation of the enzymatic cascade and subsequently to aflatoxin biosynthesis in response to oxidative stress^95^. Therefore, the expression of genes involved in aflatoxin biosynthesis is expected to decrease under low-oxygen conditions. As a result, the presence of the low-regulated lncRNAs identified under high CO_2_ concentration (such as lnc50 associated with Afl-milR-5) may be attributed to the regulation of related genes, particularly transcription factors. The results of the current study suggest that the presence of differentially expressed lncRNAs in *A. flavus*, often the down-regulated lncRNAs, may play a regulatory role in the expression of genes associated with cell survival, maintenance of metabolism, cell respiration, and control of aflatoxin biosynthesis in response to a_w_, CO_2_ concentration, and temperature changes.

### Prediction of lncRNAs localizations

**Figure 5:**
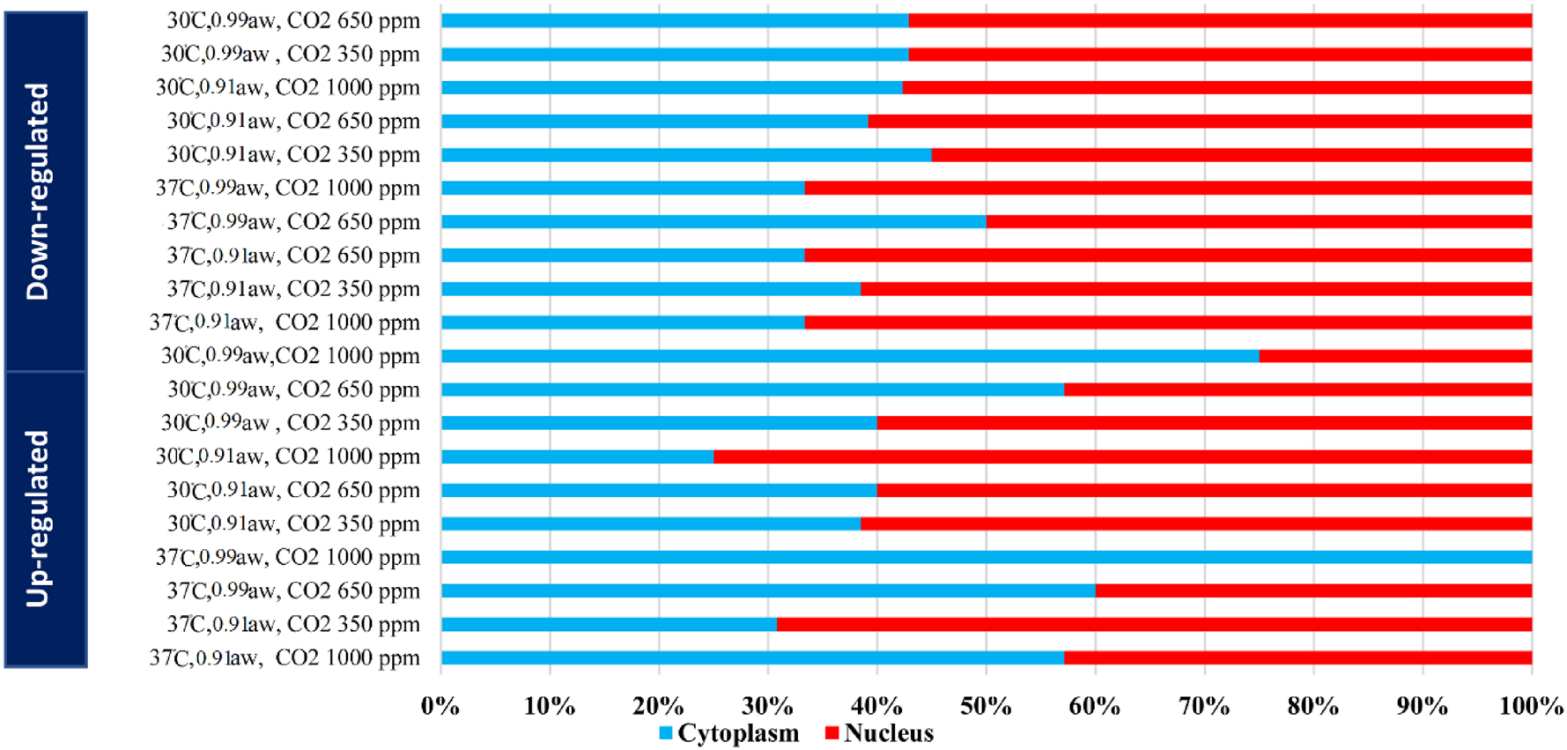
The subcellular localization of *A. flavus* lncRNAs under stress conditions.

Identifying the subcellular localization of lncRNA is a highly informative approach to understanding its biological function. While a large proportion of mRNAs are specifically localized in different cytoplasmic domains^101^, lncRNAs are variably localized in chromatin, cytoplasm, nucleus, and nucleoplasm^102^. The function of lncRNAs is closely related to their interaction with nucleic acids and RNA-binding proteins. Depending on the localization of lncRNAs and their specific interactions with proteins, RNA, and DNA, they can affect the modulation of chromatin function, the assembly of membrane-less nuclear bodies, the stability and translation of mRNAs, and signaling pathways^103^. The functions of cytoplasmic lncRNAs include maintaining cytoplasmic structure, promoting cellular organelle activity, and defining cytoplasmic domains. Cytoplasmic lncRNAs may also be associated with the modulation of cellular signaling pathways, sponging of cytosolic factors, protein turnover, RNA-binding protein (RBP) decay, microRNA decay, protein stability, mRNA turnover, and translation. Additionally, cytoplasmic lncRNA-associated ribonucleoprotein complexes (lncRNPs) may control essential events in the cytoplasm for cellular survival and maintenance of cell structure and function, such as protein turnover and its localization, mRNA stability, availability of cytoplasm ic-related factors, and scaffolding of proteins operating in a common signaling pathway^104,105^. Nuclear lncRNAs have important regulatory functions related to gene organization, transcription control, and subcellular structures. Recently, several functions of nucleus-specific lncRNAs have been revealed, including epigenetic control of transcriptional programs, the scaffolding of chromosomes, alternative splicing, and chromatin remodeling^106^. Studies have confirmed that lncRNPs associated with the nucleus play important roles in the functional integrity and structural maintenance of the nucleus. In the current study, it was predicted that the up-and down-regulated lncRNAs might be frequently localized in the nucleus under stress conditions, whereas some lncRNAs, especially the down-regulated lncRNAs under 30 °C, a_w_: 0.99, and CO_2_: 1000 ppm, and up-regulated lncRNAs under 37 °C, a_w_: 0.99, and CO_2_: 1000 ppm, might be located in the cytoplasm (Fig. 5). As a result, it was hypothesized that most of the up-regulated lncRNAs under high CO_2_ concentration stress may play important roles in regulating and modulating cytoplasmic functions, while the majority of up-and down-regulated lncRNAs, especially under water stress (a_w_: 0.91), may have regulatory roles in nuclear functions as mentioned above.

## Conclusion

In general, the results of the target analysis of lncRNAs in *A. flavus* have shown that lncRNAs, particularly the down-regulated ones, can indirectly control the down- and up-regulation of genes contributing to aflatoxin biosynthesis, maintenance of cell metabolism (e.g., proline), respiratory activity, and cellular survival under stressful conditions. The findings of the present study not only indicate the presence of lncRNAs in *A. flavus* but also serve as a valuable reference for comprehending the molecular mechanisms associated with cellular metabolism and aflatoxin production in stressful conditions.

## Materials and methods

**Figure 6:**
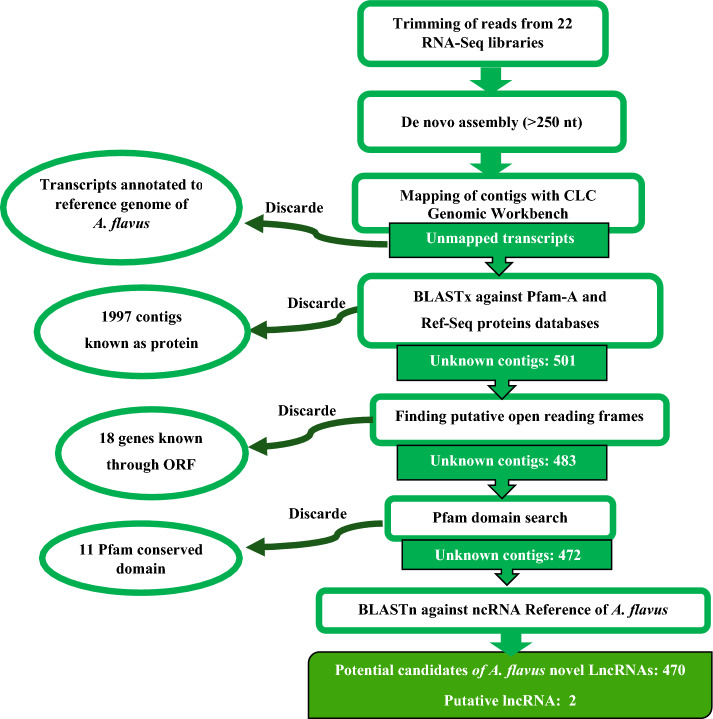
The pipeline flowchart of lncRNAs discovery.

The pipeline flowchart of the lncRNAs discovery in *A. flavus* is shown in Fig. 6.

### Preparation of RNA-Seq data

From the previous study, 22 raw RNA-Seq data sets for *A. flavus* in response to a_w_, CO_2_ concentration, and temperature changes were downloaded from the Sequence Read Archive, with Bioproject number ID: PRJNA380582 (http://www.ncbi.nlm.nih.gov/sra)^64^. The raw data were trimmed using CLC Genomic Workbench version 20 (QIAGEN). Reads with a quality score limit of 0.05 and a maximum number of 2 ambiguities were filtered for further analysis.

### Discovery of *A. flavus* lncRNAs

The de novo assembly algorithm of CLC Genomic Workbench version 20 was utilized to generate simple contig sequences, with a minimum contig length of 250, from trimmed transcripts (22 libraries). De novo assembly was performed for all samples collectively using default parameters (word size:20 and automatic bubble size) to generate transcript candidates in *A. flavus*. The resulting contigs were aligned against the most recent version of the *A. flavus* genome, including both non-chromosomal and DNA chromosomal sequences. The alignment took into account the following parameters: mismatch cost: 2, insertion cost: 3, deletion cost: 3, length fraction: 0.8, and similarity fraction: 0.8. The reference genomes and gene annotation for *A. flavus* can be found at https://www.ncbi.nlm.nih.gov (*Aspergillus flavus* NRRL3357 (GCA_014117465.1) with accession: PRJNA727281 submitted by the University of Georgia, and at http://fungi.ensembl.org. During the data analysis, unmapped reads were retained for subsequent mapping after each mapping step. Genes annotated as known *A. flavus* genes were discarded, while unannotated transcripts were filtered out for further analysis. Pfam-A, a protein domain database, and protein RefSeq for all organisms were downloaded from https://pfam.xfam.org and https://www.ncbi.nlm.nih.gov/refseq, respectively. To find other known proteins in the contig sequences, the BLASTx algorithm of CLC Genomic Workbench was employed, using the Pfam-A and Protein RefSeq databases, with the following parameters: query genetic code: 1 standard, number of threads: 20, expect: 0.0001, word size: 3, matrix: BLOSUM62, gap cost: existence 11, extension 1, and a maximum number of hit sequences: 5. Following the BLAST analysis, 501 sequences that were known and not present in the protein databases were selected. These sequences were then analyzed to find putative open reading frames (ORFs) using http://cpc2.gao-lab.org (reverse strands were also checked), and 18 known genes were discarded. Subsequently, the remaining 483 sequences were translated into six frames, and the Pfam domain search algorithm of CLC Genomic Workbench, along with the Pfam-A v35 database, was used to identify 11 conserved protein domains. Finally, a total of 472 sequences were identified as *A. flavus* lncRNAs. The ncRNAs of *A. flavus* were downloaded from https://fungi.ensembl.org and used as a reference for lncRNAs, after excluding sequences smaller than 250 nt. The BLASTn algorithm of CLC Genomic Workbench was applied to identify novel lncRNAs within the 472 filtered sequences, using the lncRNAs reference, resulting in the discovery of 470 novel lncRNAs.

### Analysis of lncRNAs expression

Expression analyses were performed for each treatment by mapping trimmed reads against 472 lncRNAs from the previous analysis as a reference. The considered parameters included: length fraction = 0.8, similarity fraction = 0.8, mismatch cost = 2, insertion cost = 3, deletion cost = 3, and the maximum number of hits for a read = 10. Expression values were determined as RPKM. The setup experiment was performed using the microarray analysis algorithm of CLC Genomic Workbench. Then, Empirical DEG was used for statistical analysis to identify lncRNAs that were highly up- or down-regulated during a_w_, CO_2_ concentration, and temperature stress. This assay compares a single treatment to a control (37 °C, a_w_: 0.99, CO_2_: 350 ppm) with a total count filter cutoff of 5.0. Volcano plots were constructed to show the most differentially expressed lncRNAs that were up-regulated (fold change > 2, p-values < 0.05) and down-regulated (fold change < -2, p-values < 0.05) compared to the control.

### Prediction of target genes

The regulatory mechanisms of lncRNAs were investigated to target the protein-coding genes located within 10 000 bp downstream or upstream regions from the lncRNAs. Subsequently, the functions of the protein-coding genes likely to be affected by the lncRNAs were determined using GO analysis.

### Prediction of lncRNA interaction with milRNAs

The miRNA data from *A. flavus* was obtained according to Bai et al. (2015). The interaction between miRNAs and lncRNAs was predicted using the psRNATarget analysis server (2017 Update) (https://www.zhaolab.org/psRNATarget/) by submitting lncRNAs as targets and miRNAs as queries. Generally, the following settings were considered: number of top targets: 200, the penalty for G: U pair: 0.5, extra weight in seed region: 1.5, number of mismatches allowed in seed region: 2, the penalty for the opening gap: 2, expectation: 5, the penalty for other mismatches: 1, seed region: 2–13 NT, HSP size: 19, the penalty for extending gap: 0.5, and translation inhibition range: 10 NT-11 NT. Interaction networks of miRNAs with lncRNAs and target proteins were constructed using Cytoscape (version 3.9.1). The nodes in this network consisted of miRNAs, while proteins and lncRNAs acted as targets of miRNAs.

### Prediction of subcellular localizations of lncRNAs

The subcellular localizations of *A. flavus* lncRNAs were based on lightGBM at http://www.biolscience.cn/LightGBM_LncLoc/.

### Ethics approval

This study does not involve any human or animal testing.

### Consent to participate

All authors are willing to publish this manuscript and they are willing to contribute to this manuscript.

## Data availability

Even though adequate data have been given in the form of tables and figures, all authors declare that if more data are required, then the data will be provided on a request basis. Although sufficient data have been provided in the form of tables and figures, all authors state that data will be provided upon request if further data are needed. The raw data used were SRR5381755, SRR5381756, SRR5381758, SRR5381761, SRR5381764, SRR5381765, SRR5381766, SRR5381767, SRR5381768, SRR5381769, SRR5381770, SRR5381771, SRR5381772, SRR5381773, SRR5381774, SRR5381775, SRR5381776, SRR5381777, SRR5381778, SRR5381779, SRR5381780, and SRR5381781. These data are available at http://www.ncbi.nlm.nih.gov/sra.

## Supplementary Information

Supplementary Information.
