# Supplementary material for: Discovery of long non-coding RNAs in Aspergillus flavus response to water activity, CO2 concentration, and temperature changes
Source: Sci Rep. 2023 Jun 26;13:10330. doi: 10.1038/s41598-023-37236-4 (PMC10293223; doi:10.1038/s41598-023-37236-4)
Supplement: Supplementary file 1 — Supplementary Information. [file 41598_2023_37236_MOESM1_ESM.docx]

Supplementary file 1

Quality control results for RNA-Seq of *A. flavus* in different treatments

|  | **Accession number** | **Sequence length** | **Read count** | **GC (%)** | **PHRED score** | **Description** |
| --- | --- | --- | --- | --- | --- | --- |
| 1 | SRR5381755 | 100 | 13 640 195 | 45 | 38 | 37˚C, a_w_: 0.99, CO_2_: 1000 ppm |
| 2 | SRR5381765 | 100 | 14 324 958 | 51 | 37 | 37˚C, a_w_: 0.91, CO_2_: 350 ppm |
| 3 | SRR5381766 | 99 | 3 563 776 | 53 | 37 | 37˚C, a_w_: 0.91, CO_2_: 350 ppm |
| 4 | SRR5381758 | 100 | 11 049 623 | 43 | 38 | 37˚C, a_w_: 0.99, CO_2_: 350 ppm |
| 5 | SRR5381761 | 100 | 8 785 209 | 46 | 38 | 37˚C, a_w_:0.91, CO_2_: 1000 ppm |
| 6 | SRR5381771 | 100 | 13 280 427 | 49 | 37 | 30˚C, a_w_: 0.99, CO_2_: 350 ppm |
| 7 | SRR5381756 | 100 | 18 205 164 | 45 | 38 | 37˚C, a_w_:0.91, CO_2_: 350 ppm |
| 8 | SRR5381767 | 100 | 13 497 209 | 53 | 37 | 37˚C, a_w_: 0.91, CO_2_: 350 ppm |
| 9 | SRR5381768 | 100 | 16 326 872 | 48 | 37 | 30˚C, a_w_: 0.99, CO_2_: 1000 ppm |
| 10 | SRR5381764 | 100 | 18 567 617 | 48 | 37 | 37˚C, a_w_: 0.91, CO_2_: 650 ppm |
| 11 | SRR5381769 | 100 | 26 179 886 | 46 | 37 | 30˚C, a_w_: 0.99, CO_2_: 650 ppm |
| 12 | SRR5381772 | 100 | 12 341 923 | 46 | 37 | 30˚C, a_w_: 0.99, CO_2_: 350 ppm |
| 13 | SRR5381776 | 100 | 11 351 674 | 52 | 37 | 30˚C, a_w_: 0.91, CO_2_: 650 ppm |
| 14 | SRR5381770 | 100 | 14 947 011 | 46 | 37 | 30˚C, a_w_: 0.99, CO_2_: 650 ppm |
| 15 | SRR5381773 | 100 | 16 327 251 | 52 | 37 | 30˚C, a_w_:0.91, CO_2_: 1000 ppm |
| 16 | SRR5381777 | 100 | 13 348 642 | 52 | 37 | 30˚C, a_w_: 0.91, CO_2_: 650 ppm |
| 17 | SRR5381775 | 100 | 11 755 115 | 52 | 37 | 30˚C, a_w_: 0.91, CO_2_: 1000 ppm |
| 18 | SRR5381774 | 100 | 15 258 103 | 53 | 37 | 30˚C, a_w_: 0.91, CO_2_: 1000 ppm |
| 19 | SRR5381779 | 100 | 19 496 071 | 52 | 37 | 30˚C, a_w_: 0.91, CO_2_: 350 ppm |
| 20 | SRR5381778 | 100 | 16 123 361 | 48 | 37 | 30˚C, a_w_:0.91, CO_2_: 650 ppm |
| 21 | SRR5381780 | 100 | 18 016 486 | 48 | 37 | 30˚C, a_w_: 0.91, CO_2_: 350 ppm |
| 22 | SRR5381781 | 100 | 17 547 803 | 53 | 37 | 30˚C, a_w_:0.91, CO_2_: 350 ppm |

Supplementary file 2

Top 10 up-regulated LncRNAs of *A. flavus* in response to a_w_, CO_2_ concentration, and temperature changes.

| **Accession No.** | **Fold change** | **Tagwise dispersions P-value (0.05)** | **Description** | **Accession No.** | **Fold change** | **Tagwise dispersions P-value (0.05)** | **Description** |
| --- | --- | --- | --- | --- | --- | --- | --- |
| lnc193 | 651.8421 | 0.001892 | **37** ˚**C, a_w_ 0.99, CO_2_ 350 ppm vs 30** ˚**C, a_w_ 0.91, CO_2_ 350 ppm** | lnc193 | 681.6842 | 0.001661 | **37** ˚**C, a_w_ 0.99, CO_2_ 350 ppm vs 30** ˚**C, a_w_ 0.91, CO_2_ 650 ppm** |
| lnc124 | 532.6844 | 0.002144 |  | lnc124 | 561.6802 | 0.001844 |  |
| lnc376 | 417.4228 | 0.004729 |  | lnc85 | 519.9963 | 0.002447 |  |
| lnc86 | 392.6157 | 0.006419 |  | lnc376 | 452.6881 | 0.003764 |  |
| lnc85 | 327.8681 | 0.005439 |  | lnc44 | 380.2407 | 0.013488 |  |
| lnc44 | 302.9707 | 0.023071 |  | lnc86 | 340.0413 | 0.009717 |  |
| lnc174 | 248.921 | 0.037365 |  | lnc181 | 334.4393 | 0.005568 |  |
| lnc181 | 247.5287 | 0.014355 |  | lnc174 | 281.597 | 0.029718 |  |
| lnc148 | 245.5093 | 0.020313 |  | lnc26 | 236.4829 | 0.041997 |  |
| lnc26 | 240.7863 | 0.040975 |  | lnc148 | 218.9602 | 0.026767 |  |
| lnc124 | 458.4504 | 0.003376 | **37** ˚**C, a_w_ 0.99, CO_2_ 350 ppm vs 30** ˚**C, a_w_ 0.91, CO_2_ 1000 ppm** | lnc33 | 2317.404 | 0.00294 | **37** ˚**C, a_w_ 0.99, CO_2_ 350 ppm vs 30** ˚**C, a_w_ 0.99, CO_2_ 350 ppm** |
| lnc193 | 455.1973 | 0.002855 |  | lnc14 | 411.0152 | 0.006499 |  |
| lnc85 | 419.7783 | 0.005073 |  | lnc86 | 267.0816 | 0.02015 |  |
| lnc376 | 330.7881 | 0.009307 |  | lnc148 | 196.8199 | 0.039128 |  |
| lnc174 | 319.6033 | 0.023733 |  | lnc193 | 190.8157 | 0.039031 |  |
| lnc86 | 239.5138 | 0.024347 |  | lnc44 | 173.0119 | 0.041134 |  |
| lnc148 | 211.9398 | 0.029083 |  | lnc340 | 144.9297 | 0.048617 |  |
| lnc181 | 190.9207 | 0.03107 |  | lnc336 | 129.2926 | 0.048926 |  |
| lnc336 | 177.2967 | 0.043259 |  | lnc293 | 14.03517 | 0.003243 |  |
| lnc340 | 145.1683 | 0.044387 |  | lnc31 | 11.9292 | 0.037561 |  |
| lnc33 | 2277.398 | 0.003028 | **37** ˚**C, a_w_ 0.99, CO_2_ 350 ppm vs 30** ˚**C, a_w_ 0.99, CO_2_ 650 ppm** | lnc124 | 590.7429 | 0.001595 | **37** ˚**C, a_w_ 0.99, CO_2_ 350 ppm vs 37**˚**C, a_w_ 0.91, CO_2_ 350 ppm** |
| lnc29 | 286.4404 | 0.037069 |  | lnc85 | 394.9339 | 0.002923 |  |
| lnc174 | 247.0142 | 0.041956 |  | lnc193 | 371.371 | 0.005516 |  |
| lnc217 | 13.3647 | 0.032468 |  | lnc376 | 367.6641 | 0.007247 |  |
| lnc293 | 11.68389 | 0.005593 |  | lnc44 | 292.5097 | 0.024957 |  |
| lnc31 | 9.791126 | 0.047787 |  | lnc181 | 282.5407 | 0.0097 |  |
| lnc216 | 6.545453 | 0.039333 |  | lnc174 | 243.4651 | 0.038288 |  |
| None of the LncRNAs showed significant expression | | | **37** ˚**C, a_w_ 0.99, CO_2_ 350 ppm vs 30** ˚**C, a_w_ 0.99, CO_2_ 1000 ppm** | lnc86 | 236.3345 | 0.026215 |  |
|  |  |  |  | lnc336 | 231.4704 | 0.020877 |  |
|  |  |  |  | lnc125 | 157.6252 | 0.045221 |  |
| lnc124 | 867.003 | 0.000811 | **37** ˚**C, a_w_ 0.99, CO_2_ 350 ppm vs 37** ˚**C, a_w_ 0.91, CO_2_ 650 ppm** | lnc33 | 395.5394 | 0.043067 | **37** ˚**C, a_w_ 0.99, CO_2_ 350 ppm vs 37** ˚**C, a_w_ 0.99, CO_2_ 650 ppm** |
| lnc376 | 456.7911 | 0.006209 |  | lnc44 | 395.5394 | 0.017888 |  |
| lnc193 | 365.6329 | 0.009829 |  | lnc12 | 378.3855 | 0.012476 |  |
| lnc86 | 274.4746 | 0.025194 |  | lnc85 | 258.3083 | 0.01971 |  |
| lnc44 | 183.3164 | 0.044892 |  | lnc17 | 224.0005 | 0.027262 |  |
| lnc85 | 183.3164 | 0.048843 |  | lnc148 | 155.385 | 0.044357 |  |
| lnc336 | 168.1234 | 0.034695 |  | lnc31 | 16.04521 | 0.023317 |  |
| lnc256 | 22.41369 | 0.006943 |  |  |  |  |  |
| lnc124 | 539.3161 | 0.00361 | **37** ˚**C, a_w_ 0.99, CO_2_ 350 ppm vs 37** ˚**C, a_w_ 0.91, CO_2_ 1000 ppm** | lnc26 | 381.7975 | 0.022738 | **37** ˚**C, a_w_ 0.99, CO_2_ 350 ppm vs 37** ˚**C, a_w_ 0.99, CO_2_ 1000 ppm** |
| lnc174 | 308.6092 | 0.031513 |  | lnc3 | 286.5981 | 0.031187 |  |
| lnc17 | 289.3836 | 0.029597 |  | lnc17 | 215.1986 | 0.030502 |  |
| lnc85 | 250.9325 | 0.022174 |  | lnc124 | 191.3988 | 0.028839 |  |
| lnc14 | 193.2557 | 0.031885 |  | lnc12 | 167.5989 | 0.044749 |  |
| lnc148 | 154.8046 | 0.044357 |  |  |  |  |  |
| lnc31 | 11.59481 | 0.040329 |  |  |  |  |  |

Supplementary file 3

Top 10 down-regulated LncRNAs of *A. flavus* in response to a_w_, CO_2_ concentration, and temperature changes.

| **Accession No.** | **Fold change** | **Tagwise dispersions**  **P-value (0.05)** | **Description** | **Accession No.** | **Fold change** | **Tagwise dispersions**  **P-value (0.05)** | **Description** |
| --- | --- | --- | --- | --- | --- | --- | --- |
| lnc24 | -1935.04 | 2.55E-05 | **37** ˚**C, a_w_ 0.99, CO_2_ 350 ppm vs 30** ˚**C, a_w_ 0.91, CO_2_ 350 ppm** | lnc24 | -1935.04 | 2.55E-05 | **37** ˚**C, a_w_ 0.99, CO_2_ 350 ppm vs 30** ˚**C, a_w_ 0.91, CO_2_ 650 ppm** |
| lnc49 | -174.351 | 0.000424 |  | lnc43 | -378.374 | 3.03E-05 |  |
| lnc57 | -142.515 | 0.012921 |  | lnc108 | -284.03 | 0.011095 |  |
| lnc43 | -48.126 | 0.000131 |  | lnc5 | -55.984 | 7.99E-05 |  |
| EFT00053849665 | -32.4247 | 4.39E-06 |  | EFT00053849665 | -47.0552 | 5.56E-07 |  |
| lnc48 | -29.1625 | 2.92E-05 |  | lnc48 | -33.5367 | 1.5E-05 |  |
| EFT00053849670 | -19.5783 | 4.09E-05 |  | lnc59 | -32.9839 | 0.000149 |  |
| lnc240 | -17.3522 | 0.017788 |  | lnc240 | -27.5902 | 0.01264 |  |
| lnc79 | -16.952 | 0.003308 |  | lnc19 | -27.5489 | 0.018589 |  |
| lnc59 | -16.5326 | 0.001551 |  | EFT00053849670 | -26.8671 | 7.14E-06 |  |
| lnc24 | -1935.04 | 2.55E-05 | **37** ˚**C, a_w_ 0.99, CO_2_ 350 ppm vs 30** ˚**C, a_w_ 0.91, CO_2_ 1000 ppm** | lnc108 | -284.03 | 0.045303 | **37** ˚**C, a_w_ 0.99, CO_2_ 350 ppm vs 30** ˚**C, a_w_ 0.99, CO_2_ 350 ppm** |
| lnc43 | -378.374 | 3.03E-05 |  | lnc24 | -254.76 | 0.001782 |  |
| lnc108 | -284.03 | 0.011095 |  | lnc187 | -189.687 | 0.002274 |  |
| lnc131 | -142.515 | 0.033139 |  | lnc163 | -142.515 | 0.011595 |  |
| lnc49 | -110.023 | 0.001393 |  | lnc19 | -39.8463 | 0.034758 |  |
| EFT00053849665 | -88.2351 | 1.51E-08 |  | lnc59 | -31.6576 | 0.001709 |  |
| lnc48 | -87.2738 | 1.29E-07 |  | lnc5 | -29.9546 | 0.003961 |  |
| lnc5 | -86.5726 | 1.86E-05 |  | lnc103 | -21.306 | 0.001184 |  |
| lnc95 | -57.2059 | 0.003894 |  | lnc49 | -19.9841 | 0.040139 |  |
| lnc79 | -55.7985 | 7.26E-05 |  | EFT00053849665 | -17.4653 | 0.000976 |  |
| lnc59 | -18.2459 | 0.009341 |  | lnc59 | -661.404 | 0.005283 | **37** ˚**C, a_w_ 0.99, CO_2_ 350 ppm vs 37** ˚**C, a_w_ 0.99, CO_2_ 650 ppm** |
| lnc49 | -472.717 | 0.00435 | **37** ˚**C, a_w_ 0.99, CO_2_ 350 ppm vs 30** ˚**C, a_w_ 0.99, CO_2_ 650 ppm** | lnc80 | -378.374 | 0.004364 |  |
| lnc108 | -284.03 | 0.045303 |  | lnc84 | -236.858 | 0.032286 |  |
| lnc57 | -142.515 | 0.037369 |  | lnc156 | -189.687 | 0.026006 |  |
| lnc240 | -51.0771 | 0.018227 |  | lnc62 | -189.687 | 0.039714 |  |
| lnc59 | -39.2489 | 0.001709 |  | lnc69 | -189.687 | 0.010288 |  |
| lnc43 | -20.6966 | 0.006632 |  | lnc163 | -142.515 | 0.037125 |  |
| lnc62 | -18.6432 | 0.01142 |  | lnc5 | -30.9855 | 0.04144 |  |
| lnc106 | -17.0226 | 0.027727 |  | lnc59 | -661.404 | 0.005283 | **37** ˚**C, 0.99 a_w_, CO_2_ 350 ppm vs 37** ˚**C, 0.91 a_w_, CO_2_ 1000 ppm** |
| lnc50 | -14.9911 | 0.006123 |  | lnc43 | -378.374 | 0.015288 |  |
| lnc52 | -13.7193 | 0.021529 |  | lnc27 | -19.3112 | 0.030323 |  |
| lnc8 | -4199.28 | 4.99E-05 | **37** ˚**C, a_w_ 0.99, CO_2_ 350 ppm vs 30** ˚**C, a_w_ 0.99, CO_2_ 1000 ppm** | lnc131 | -142.515 | 0.029581 | **37** ˚**C, a_w_ 0.99, CO_2_ 350 ppm vs 37**˚**C, a_w_ 0.91, CO_2_ 350 ppm** |
| lnc6 | -944.434 | 0.001312 |  | lnc58 | -95.3434 | 0.018793 |  |
|  |  |  |  | lnc43 | -76.9947 | 0.000335 |  |
|  |  |  |  | lnc5 | -29.8441 | 0.000617 |  |
| lnc15 | -661.404 | 0.00917 |  | lnc59 | -27.6972 | 0.000374 |  |
| lnc59 | -661.404 | 0.024412 |  | lnc24 | -26.602 | 0.009578 |  |
| lnc21 | -331.202 | 0.016864 |  | lnc240 | -14.8662 | 0.025462 |  |
| lnc50 | -284.03 | 0.046809 |  | lnc49 | -11.1622 | 0.04557 |  |
| lnc216 | -236.858 | 0.044341 |  | lnc79 | -11.096 | 0.011229 |  |
| lnc276 | -7.93346 | 0.015163 |  | lnc48 | -10.4607 | 0.002651 |  |
| lnc84 | -236.858 | 0.032286 | **37** ˚**C, a_w_ 0.99, CO_2_ 350 ppm vs 37** ˚**C, a_w_ 0.99, CO_2_ 1000 ppm** | lnc43 | -378.374 | 0.015288 | **37**˚**, a_w_ 0.99, CO_2_ 350 ppm vs 37**˚**^0^C, a_w_0.91, CO_2_ 650 ppm** |
| lnc187 | -189.687 | 0.044775 |  | lnc50 | -284.03 | 0.007928 |  |
| lnc163 | -142.515 | 0.037125 |  |  |  |  |  |
| lnc95 | -142.515 | 0.048824 |  |  |  |  |  |
| lnc27 | -23.3154 | 0.0237 |  |  |  |  |  |
| lnc9 | -11.4529 | 0.047288 |  | lnc59 | -21.0732 | 0.038163 |  |

Supplementary file 4

Predicted interactions between lncRNAs and milRNAs

| **Treatment** | **Targets of lncRNAs** | |
| --- | --- | --- |
|  | **Significant Up- regulated lncRNAs** | **Significant Down- regulated lncRNAs** |
|  | **(Target milRNAs, target proteins)** | **(Target milRNAs, target proteins)** |
| **30 ^˚^C, a_w_: 0.91, CO_2_: 350 ppm** | **lnc52** (Afl-milR-15; AFL2T_10468) | **EFT00053849670 (**Afl-milR-32; AFL2T_02855) |
|  |  | **EFT00053849665** (Afl-milR-42; AFL2T_05705) |
|  |  | **lnc59 (**Afl-milR-48; AFL2T_02069) |
|  |  | **lnc48** (Afl-milR-6; AFL2T_04445, AFL2T_08349, AFL2T_09091, AFL2T_10492) |
| **30 ^˚^C, a_w_: 0.91, CO_2_: 650 ppm** | **lnc52** (Afl-milR-15; FL2T_10468) | **EFT00053849670 (**Afl-milR-32; AFL2T_02855) |
|  |  | **EFT00053849665** (Afl-milR-42; AFL2T_05705) |
|  |  | **lnc59 (**Afl-milR-48; AFL2T_02069) |
|  |  | **lnc48** (Afl-milR-6; AFL2T_04445, AFL2T_08349, AFL2T_09091, AFL2T_10492) |
| **30 ^˚^C, a_w_: 0.91, CO_2_: 1000 ppm** | **lnc52** (Afl-milR-15; AFL2T_10468) | **EFT00053849670 (**Afl-milR-32; AFL2T_02855) |
|  |  | **EFT00053849665** (Afl-milR-42, AFL2T_05705) |
|  |  | **lnc59 (**Afl-milR-48; AFL2T_02069) |
|  |  | **lnc48** (Afl-milR-6; AFL2T_04445, AFL2T_08349, AFL2T_09091, AFL2T_10492) |
| **30 ^˚^C, a_w_: 0.99, CO_2_: 350 ppm** |  | **EFT00053849670 (**Afl-milR-32; AFL2T_02855) |
|  |  | **EFT00053849665** (Afl-milR-42; AFL2T_05705) |
|  |  | **lnc59 (**Afl-milR-48; AFL2T_02069) |
|  |  | **lnc48** (Afl-milR-6; AFL2T_04445, AFL2T_08349, AFL2T_09091, AFL2T_10492) |
| **30 ^˚^C, a_w_: 0.99, CO_2_: 650 ppm** | **lnc216** (Afl-milR-25; AFL2T_00996, AFL2T_08541) | **lnc52** (Afl-milR-15; AFL2T_10468) |
|  |  | **lnc4 (**Afl-milR-39; AFL2T_01927) |
|  |  | **lnc62 (**Afl-milR-4; AFL2T_09890) |
|  |  | **lnc59 (**Afl-milR-48; AFL2T_02069) |
|  |  | **lnc50 (**Afl-milR-5; AFL2T_02174, AFL2T_04761, AFL2T_10314) |
| **30 ^˚^C, a_w_: 0.99, CO_2_: 1000 ppm** |  | **lnc216** (Afl-milR-25; AFL2T_00996, AFL2T_08541) |
|  |  | **lnc59 (**Afl-milR-48; AFL2T_02069) |
|  |  | **lnc50 (**Afl-milR-5; AFL2T_02174, AFL2T_04761, AFL2T_10314) |
|  |  | **lnc21** (Afl-milR-6; AFL2T_04445, AFL2T_08349, AFL2T_09091, AFL2T_10492- Afl-milR-15; AFL2T_10468) |
|  |  | **lnc52** (Afl-milR-15; AFL2T_10468) |
| **37 ^˚^C, a_w_: 0.91, CO_2_: 350 ppm** |  | **EFT00053849670 (**Afl-milR-32; AFL2T_02855) |
|  |  | **EFT00053849665** (Afl-milR-42; AFL2T_05705) |
|  |  | **lnc59 (**Afl-milR-48; AFL2T_02069) |
|  |  | **lnc48** (Afl-milR-6; AFL2T_04445, AFL2T_08349, AFL2T_09091, AFL2T_10492) |
| **37 ^˚^C, a_w_: 0.91, CO_2_: 650 ppm** |  | **lnc59 (**Afl-milR-48; AFL2T_02069) |
|  |  | **lnc50 (**Afl-milR-5; AFL2T_02174, AFL2T_04761, AFL2T_10314) |
| **37 ^˚^C, a_w_: 0.91, CO_2_: 1000 ppm** |  | **lnc59 (**Afl-milR-48; AFL2T_02069) |
| **37 ^˚^C, a_w_: 0.99, CO_2_: 650 ppm** |  | **lnc59 (**Afl-milR-48; AFL2T_02069) |
|  |  | **lnc62 (**Afl-milR-4, AFL2T_09890) |
| **37 ^˚^C, a_w_: 0.99, CO_2_: 1000 ppm** | **lnc3** (Afl-milR-33; AFL2T_08520, AFL2T_03607, AFL2T_08912) | **lnc9** (Afl-milR-23; AFL2T_08112, AFL2T_09480) |
|  |  |  |
| AFL2T_10314, AFL2T_02069, AFL2T_03607, AFL2T_02174, AFL2T_04445, AFL2T_09890, AFL2T_00996 and AFL2T_08541: hypothetical protein. | |  |
| AFL2T_01927: hypothetical protein, acetate--CoA ligase. |  |  |
| AFL2T_02855: permease. |  |  |
| AFL2T_04761: pH-response regulator protein palA/RIM20. |  |  |
| AFL2T_05705: serine-rich protein, putative. |  |  |
| AFL2T_08112: ankyrin repeat protein - pyridine nucleotide-disulfide oxidoreductase. | |  |
| AFL2T_08349: Temperature dependent protein affecting M2 dsRNA replication. |  |  |
| AFL2T_08520: NRPS-like enzyme, gamma glutamyl transpeptidase (UstH), which is responsible for ustiloxin B biosynthesis, gamma-glutamyltransferase-selenocysteine lyase. | | |
| AFL2T_09091: cytochrome P450, putative. |  |  |
| AFL2T_09480: pleiotropic drug resistance proteins (PDR1-15), ABC superfamily-ATP-binding cassette transporter-ATP-binding cassette transporter, putative-pleiotropic drug resistance protein. | | |
| AFL2T_10468: ubiquinone biosynthesis protein coq9. |  |  |
| AFL2T_10492: NIF3 NGG1p interacting factor 3 -3-ketosteroid-delta-1-dehydrogenase, putative. | |  |
